# Climate adaptation and speciation: particular focus on reproductive barriers in *Ficedula* flycatchers

**DOI:** 10.1111/eva.12276

**Published:** 2015-06-30

**Authors:** Anna Qvarnström, Murielle Ålund, S. Eryn McFarlane, Päivi M. Sirkiä

**Affiliations:** ^1^ Animal Ecology/Department of Ecology and GeneticsUppsala UniversityUppsalaSweden; ^2^ Finnish Museum of Natural HistoryZoology UnitUniversity of HelsinkiHelsinkiFinland

**Keywords:** ecological speciation, genetic incompatibilities, natural selection, personality, sexual selection, speciation genomics, thermal adaptation

## Abstract

Climate adaptation is surprisingly rarely reported as a cause for the build‐up of reproductive isolation between diverging populations. In this review, we summarize evidence for effects of climate adaptation on pre‐ and postzygotic isolation between emerging species with a particular focus on pied (*Ficedula hypoleuca*) and collared (*Ficedula albicollis*) flycatchers as a model for research on speciation. Effects of climate adaptation on prezygotic isolation or extrinsic selection against hybrids have been documented in several taxa, but the combined action of climate adaptation and sexual selection is particularly well explored in *Ficedula* flycatchers. There is a general lack of evidence for divergent climate adaptation causing intrinsic postzygotic isolation. However, we argue that the profound effects of divergence in climate adaptation on the whole biochemical machinery of organisms and hence many underlying genes should increase the likelihood of genetic incompatibilities arising as side effects. Fast temperature‐dependent co‐evolution between mitochondrial and nuclear genomes may be particularly likely to lead to hybrid sterility. Thus, how climate adaptation relates to reproductive isolation is best explored in relation to fast‐evolving barriers to gene flow, while more research on later stages of divergence is needed to achieve a complete understanding of climate‐driven speciation.

## Introduction

In sexually reproducing organisms, the process of speciation is often seen as a gradual build‐up of a combination of pre‐ and postzygotic reproductive barriers between genetically diverging populations (Coyne and Orr 2004). Research on ecological speciation (Schluter 2000; Dieckmann et al. 2004; Nosil 2012) focuses on how populations become reproductively isolated from each other as a consequence of adapting to abiotic and biotic factors. Most empirical work on ecological speciation has focused on traits involved in resource use, such as beak shape in Galapagos finches and mouth structure in cichlids, in young species with few or no genetic incompatibilities (reviewed in Schluter 2000; Dieckmann et al. 2004; Nosil 2012). This bias towards young radiations, and hence a subset of sources of reproductive isolation, can partly be explained by the fact that as time passes from the original split, it becomes more difficult to establish the role of ecological adaptation in relation to other forces driving population divergence, such as genetic drift or co‐evolutionary arms races between selfish genetic elements and suppressors. However, advances in genetic sequencing techniques offer novel possibilities to make inferences about the evolutionary history of natural populations and how such histories are entwined with the rise of various sources of reproductive isolation (e.g. reviewed by Rice et al. 2011; Seehausen et al. 2014). Studying both processes of adaptation which are morphologically cryptic and possible links to the build‐up of typically slowly arising reproductive barriers (e.g. genetic incompatibilities) has therefore become more feasible than ever before. Adaptation to different climatic conditions often relies on physiological traits (reviewed by Kearney and Porter 2009) and may therefore appear morphologically cryptic. This may lead to an underestimation of the role of climate adaptation in the gradual build‐up of reproductive isolation.

By contrast, climate change has received considerable attention in relation to speciation through indirect effects on the geographical ranges of populations. Palaeoclimatic records combined with population genetic models have revealed relationships between large‐scale historical oscillations in climate and diversification rates in both animals and plants (Hewitt 1996). The general view is that these historical climatic fluctuations (e.g. cycles of ice ages during the Pleistocene) resulted in habitat fragmentation, which in turn allowed the build‐up of genetic differences among populations living in separated refugia through prohibition of gene flow between them. The geographical location of these refugia probably had a long‐term impact on species distributions. Contemporary clusters of hybrid zones where northern and southern taxa meet are likely to reflect rapid range expansions of northern species living at the southern edge of Pleistocene glaciers (e.g. Hewitt 1999). However, despite this well‐documented role of climate in speciation through the shrinking and expanding of the distribution of populations, few studies have investigated whether and how *adaptation* to different climates relates to the build‐up of reproductive isolation. This is surprising because researchers as early as Darwin (1859) and Wallace (1878) suggested that ecological adaptations in the temperate zone should be largely driven by climate. There is also a general agreement that neutral genetic change through drift is a slow process compared to changes in genes under selection (e.g. Ehrlich and Raven 1969; Slatkin 1987). Thus, climate change will affect speciation not only indirectly through changes in distribution ranges but also directly through links between divergent climate adaptation and the build‐up of reproductive isolation between populations.

**Figure 1:**
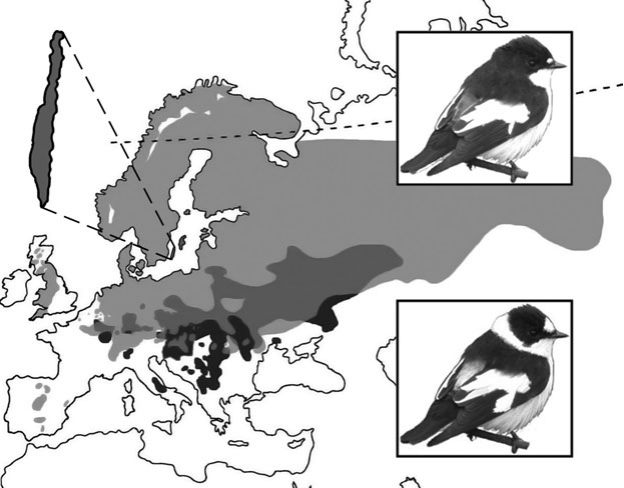
Pied and collared flycatchers. Collared (bottom right) and pied flycatchers (top right) are two small, short‐lived migratory passerines that winter in sub‐Saharan Africa and breed in Europe (Cramp and Simmons 2006). The two species diverged from their common ancestor less than one million years ago (Nadachowska‐Brzyska et al. 2013) and have probably gone through cycles of geographical isolation in separate refugia of the Mediterranean area during the ice ages followed by breeding range expansions northwards. The contemporary breeding range of pied flycatchers (light and dark grey) covers most of Europe, whereas the breeding range of collared flycatchers (black and dark grey) is restricted to warmer and more continental regions in Central and Eastern Europe and to the two Baltic islands, Gotland and Öland. Thus, differences in the overall breeding ranges of pied and collared flycatchers in Europe imply that collared flycatchers are relatively more limited by climate. Collared flycatchers therefore belong to the group of species that are expected to shift breeding range northwards in response to current climate change (Huntley et al. 2007). There are currently two contact zones: one broad hybrid zone in Central and Eastern Europe and one more isolated hybrid zone on the Baltic islands of Öland and Gotland, Sweden (both shown in dark grey). Our research is concentrated on the island of Öland that is enlarged (top left). The Swedish hybrid zone is relatively young and arose when collared flycatchers expanded their breeding range into areas where pied flycatchers were already present (Qvarnström et al. 2010). There is little divergence in size (Merilä et al. 1994), feeding techniques (Alerstam et al. 1978) or diet (Wiley et al. 2007) between the two species, leading to previous conclusions of a limited role of ecology in their speciation process. Moreover, both species breed in nest cavities (or nest boxes when provided) in deciduous forests leading to competition over nesting sites, which collared flycatchers are more likely to win (Qvarnström et al. 2010; Sætre and Sæther 2010).

**Figure 2:**
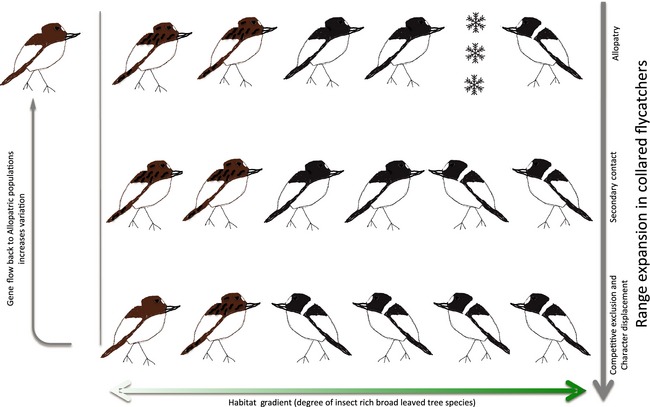
Feedback loops between evolution at different geographical regions. Pied flycatchers have a more northern breeding distribution as compared to collared flycatchers and use a comparatively overall broader niche (here illustrated in terms of habitat use). These differences set the stage for competitive interactions where the two species come into secondary contact. The two species recently came into secondary contact on the Swedish island Öland as collared flycatchers expanded their breeding range northwards. As time progresses, competitive interactions between collared and pied flycatchers lead to competitive exclusion of pied flycatchers from the preferred broadleaved deciduous forest into a more mixed habitat type (i.e. including also conifer tree species). Relatively brown pied flycatchers are often found in a more mixed forest habitat also in the absence of collared flycatchers possibly because they are less competitive than black pied flycatchers. Relatively brown pied flycatchers are favoured by selection in areas where the two species co‐occur and are found in higher frequency in the hybrid zones. Gene flow from sympatric areas may increase the frequency of brown male pied flycatchers in allopatric populations and further expand the niche use of pied flycatchers into a more mixed forest habitat type. Thus, a feedback loop from sympatric populations to allopatric pied flycatcher populations is expected to lead to increased genetic diversity in plumage coloration and niche use in allopatric populations of pied flycatchers. When collared flycatchers continue to expand their breeding distribution, the pre‐existing differences between the two species may therefore already have been augmented through gene flow. Increased asymmetry in the initial competitive ability of the two species may then further speed up competition‐driven niche separation. Flycatcher drawings by Johan Ålund.

**Figure 3:**
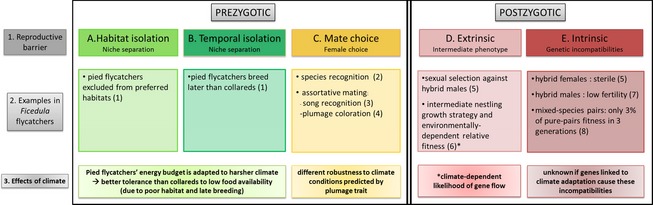
Reproductive barriers between collared and pied flycatchers at secondary contact. Collared and pied flycatchers breeding in a young contact zone on the Swedish island Öland are to a high degree reproductively isolated from each other through the action of a combination of pre‐ and postzygotic barriers. Pied flycatchers are becoming displaced from the most preferred breeding sites leading to habitat segregation (A) and on average onset their breeding later than collared flycatchers (B). Females of both species show genetically determined preferences for males of their own species (C), and differences in male plumage traits and song facilitates species assortative mating (C). However, apparent constrains in female mate choice and remaining overlap in male song and plumage traits regularly result in heterospecific pairing and the production of hybrids. Hybrid nestlings experience environmental‐dependent relative fitness in comparison with purebred nestlings (D), and male hybrids experience a disadvantage in competition over mates (D). Female hybrids appear to be completely sterile, while male hybrids experience strongly reduced fertility (E). How differences in climate adaptation relate to the various forms of reproductive isolation is specified in the figure. References: 1: Vallin et al. (2012b); 2: Sæther et al. (2007); 3: Qvarnström et al. (2006); 4: Wiley et al. (2005); 5: Svedin et al. (2008); 6: Vallin et al. (2013); 7: Ålund et al. (2013); 8: Wiley et al. (2009).

The aim of this review is to summarize current evidence for a role of climate adaptation in the build‐up of reproductive isolation. We will particularly focus on work using collared and pied flycatchers as a research model (Fig. 1). As the role of climate adaptation in the build‐up of reproductive isolation remains largely unexplored, our aim is to provide a broad overview of this research field while leaving some important aspects of the flycatcher system out, which are less relevant to the climate topic. The use of the flycatcher system as a recurrent example can be motivated by two main facts (apart from giving us the opportunity to display our own work). First, this system illustrates how climate adaptation relates to feedback loops between evolution under allopatric and sympatric conditions (Fig. 2). This example thus captures the dual effect of climate on diversification: direct effects of divergent climate adaptation on the build‐up of reproductive isolation; and indirect effect of climate on the process of speciation by affecting the distribution of populations. Second, the presence of a broad range of different sources of reproductive isolation in *Ficedula* flycatchers (Fig. 3) means that we can relate divergent climate adaptation to many different sources of reproductive isolation, which typically evolve at different rates.

We will focus our review on three main aspects of climate adaptation and speciation:

1. Climate adaptation leading to spatial and temporal isolation and/or extrinsic selection against hybrids;
2. Climate‐driven divergence in sexual display traits causing assortative mating; and
3. Climate adaptation and the build‐up of genetic incompatibilities.

We conclude that climate adaptation may play a more important role in the speciation process than is often realized. We would also like to encourage more studies on feedback loops between divergence under sympatric and allopatric conditions rather than aiming at isolating their relative importance. Our personal reflections on gender biases in science are found in Box 1.

Box 1 **Personal reflections on gender biases in science**

We are four women from different countries and backgrounds and at very different stages of our scientific careers. Anna Qvarnström is PI and has been running the flycatcher‐speciation‐project on Öland since 2001, Päivi Sirkiä is at the postdoc level studying environment‐driven mechanisms in the maintenance of phenotypic variation, and Murielle Ålund and Eryn McFarlane are both halfway through their PhD studies, studying the role of gametes in speciation and genetics of life‐history traits, respectively. Some of us have children, and we are all starting to experience/have experienced the difficulties of planning a scientific career and when to start a family, and then combining a family with a very productive scientific life, including months of field work, travels and intense working periods before important deadlines. We all agree that things have been generally improving for women over the past decades, and maybe particularly in Scandinavia, where governmental policies (e.g. equal rights to parental leave) help to move towards equal opportunities between genders. However, this movement is still slow, particularly in academia, where men are still highly overrepresented, especially for long‐term positions.

We would like to stress that it is important not to fall into the trap of seeing gender issues as some sort of tug of war between men and women. Striving for a better gender balance at all levels of organization at universities, research institutes and research agencies is instead a quality issue concerning us all. This is because a gender bias in itself creates biased expectations and prejudgements, which in turn are contra‐productive. A recent study showed that both men and women tend to consider brilliance or specific talents as typical male attributes (Leslie et al. 2015). Biases in expectations might bias our interpretations of details that might count in evaluation processes. For example, a male scientist with a well‐developed network of collaborators is often judged to have excellent management skills, while a woman in the same position is more likely to get her independence questioned.

A major issue resulting from this expectation of men to be more talented is that often fewer women apply for important positions/grants, even though they would be perfectly qualified (e.g. Nurse 2015). However, more women are needed at all positions to change these expectations and also because current attempts at enforcing equality in all decision panels results in extreme work loads for the few women who have to be member of all possible evaluation processes. We would like to encourage women to study sciences, apply for positions and grants and most importantly believe in themselves and their talent. This is the only way to prove those prejudices wrong and get a real chance to fill the gender gaps at all level of academia. The younger members of our team feel extremely inspired by the example of established women scientists. The importance of mentors and role models is recognized as a factor encouraging women to pursue scientific careers (Bonetta 2010), and the multiplication of initiatives such as this very issue of Evolutionary Applications should help women to realize that there is no real reason for them to be less successful in science than men.

## Climate adaptation leading to spatial and temporal isolation and/or extrinsic selection against hybrids

### Climate and physiology

Adaptation to climatic conditions can be seen as physiological tolerance of environmental factors such as temperature or moisture. Temperature is known to have large effects on the biochemistry of living organisms (e.g. Kingsolver 2009) and thereby influences their central life‐history traits such as growth, development and reproduction. As individuals are constrained by how much energy they can use, life‐history trade‐offs can be rethought in the context of metabolic rate as individuals' ‘decisions’ on where to allocate their energy (Stearns 1992). Metabolic rate (MR) is a measure of the energy that an individual is using at any given time, and can be broken down into different measurements such as basal metabolic rate, resting metabolic rate, field metabolic rate and maximum metabolic rate (See Glossary in Box 2). Measures of metabolic rate have indeed been found to co‐vary with traditional life‐history traits such as growth rate (Blackmer et al. 2005; Criscuolo et al. 2008) and survival (Larivee et al. 2010). A species' metabolic rate is specifically expected to relate to the geographical range it inhabits, as metabolic rate has been found to affect thermoregulation (Naya et al. 2013). Indeed, interspecific differences in metabolic rate are well predicted by latitude (Lovegrove 2003), dryness (McNab and Morrison 1963), climate (Song and Wang 2006), or net primary productivity of a species' range (Mueller and Diamond 2001). There are also studies relating metabolic rate with other important traits such as immune function (Ots et al. 2001) and animal personality (Careau et al. 2008; Biro and Stamps 2010). Ecologists therefore often use measures of metabolic rate to summarize the effects of adaptation to different climatic conditions. In short, climate adaptation has profound effects on the energy budget of organisms and is tightly interwoven with life‐history evolution.

Box 2 **Glossary**

**Allopatric speciation:** Speciation without gene flow, in geographically isolated populations.

**Basal metabolic rate (BMR):** Amount of energy needed by an individual to keep its organs functioning.

**Character displacement:** Individuals of two (related) species have accentuated differences in a trait when their breeding ranges overlap (sympatry) as compared to the value of this trait in geographically isolated populations (allopatry).

**Dobzhansky–Muller incompatibilities:** Genetic incompatibilities affecting hybrids, arising as new alleles appear in different, isolated populations (at least 2 loci), and do not function together in hybrids as they lack a co‐evolutionary history.

**Field metabolic rate (FMR or DEE):** Amount of energy used by an individual in its daily life.

**Haldane's rule:** Describes how hybrids of different sexes are differently affected by genetic incompatibilities. The heterogametic sex is generally affected first and thus tends to lose fertility and viability before the homogametic sex.

**Heterogametic:** Individuals with two different sex chromosomes, for example male mammals (XY) and female birds (ZW).

**Homogametic:** Individuals with two identical sex chromosomes, for example female mammals (XX) and male birds (ZZ).

**Maximum metabolic rate (MMR):** Total amount of energy spent by an individual under stressful conditions (e.g. harsh environment).

**Muller's ratchet:** Irreversible accumulation of deleterious mutations in chromosomes or parts of the genome/organisms that do not recombine/reproduce sexually.

**Postzygotic isolation:** Barriers to heterospecific breeding acting after fertilization, altering hybrid fitness through ecological mismatch (extrinsic) or genetic incompatibilities (intrinsic).

**Prezygotic isolation:** Barriers to heterospecific breeding acting before mating or fertilization.

**Reinforcement:** Enhanced prezygotic isolation at secondary contact between diverged populations as a result of selection against the production of unfit hybrid offspring.

**Resting metabolic rate (RMR):** BMR plus the energy needed for digestion.

**Sympatric speciation:** Speciation with gene flow, in populations living in the same geographical area, initially interbreeding to some extent.

### Climate and phenology

When organisms adapt to different climates, they also need to adjust to altered biotic factors such as selection pressures arising from variation in abundance of parasites, predators and prey. Much research on the current ongoing global climate change focuses on the effects of shifts in phenology, that is the seasonal timing of life‐history events, across different trophic levels. Fast changes in climate can lead to mismatches in phenology between trophic levels (Thackeray et al. 2010; Plard et al. 2014; Gienapp et al. 2014), which can have severe negative fitness effects on individual species. This, in turn, illustrates the importance of co‐adaptation within biotic communities living in different local climate zones. The effects of climate on adaptation *per se* have received considerable scientific attention, and this literature is far from covered by this review. Our main point is that climate adaptation has a large impact on a number of important traits, and still has received comparatively little attention in relation to speciation, especially in relation to sources of reproductive isolation that evolve relatively slowly.

### Climate, niche differentiation and reproductive isolation

Allopatric divergence in general physiological tolerance of environmental factors (e.g. temperature or moisture) and in life‐history strategies may directly lead to prezygotic isolation between populations at secondary contact. Closely related species often segregate along climate gradients (e.g. Martin 2001), suggesting that they are unable to inhabit each other's thermal niches. Different tolerance ranges may also reduce gene flow at the microhabitat level. Thermal niche segregation has, for example, been documented in brook chars within the same lake (Bertolo et al. 2011) and among whitefish morphs (*Coregonus lavaretus*; Kahilainen et al. 2014). Prezygotic isolation through segregation in timing of breeding has been studied extensively in plants (Lowry et al. 2008). For example, yellow monkey flowers (*Mimulus guttatus*) in western North America exhibit differences in flowering times, due to a chromosomal inversion (Lowry and Willis 2010). This inversion has led to a split between annual and perennial life histories in this species and, thus, reproductive isolation (Lowry and Willis 2010). Speciation through divergence in phenology may also operate at the microhabitat scale level. An appealing example is the *Howea* palm system. These palms have become reproductively isolated via differences in soil pH, where *H. forsteriana* is found in more basic soil, which heats up faster, and therefore *H. forsteriana* flowers earlier, while *H. belmoreana* prefers more acidic soil and flowers later in the season (Savolainen et al. 2006). This system illustrates how a change in microhabitat, in this case pH, can affect a life‐history trait (phenology), resulting in reproductive isolation. Prezygotic isolation through segregation in the onset of breeding is also well documented in animals including insects (e.g. Harrison 1985; Ordin et al. 2010) and amphibians (e.g. Hillis 1981). Postzygotic isolation in the form of ecologically inferior hybrids depends on access to ‘intermediate’ thermal environments. In the case of an elevational gradient between west slope cutthroat trout and introduced rainbow trout, hybrids were found to occupy an intermediate elevation and to express intermediate metabolic phenotypes, suggesting bounded hybrid superiority at intermediate elevations (Rasmussen et al. 2012). Thus, there are several studies relating climate adaptation to sources of reproductive isolation that typically evolve relatively quickly. However, one could argue that many species should converge in climate adaptation where their breeding ranges overlap making reproductive isolation unstable from a longer time perspective.

### Climate, niche differentiation and reproductive isolation in *Ficedula* flycatchers

We study the speciation process using *Ficedula* flycatchers as a model system. The current differences in breeding ranges of pied and collared flycatchers (Fig. 1) and asymmetry in ability to compete over nesting sites can be translated into a difference in how they trade off competitive ability and tolerance to more northern climate conditions. More precisely, pied flycatchers have a relatively more northern breeding distribution and are therefore more tolerant to harsh conditions in terms of, for example, coldness and food shortage but are less successful in interference competition over limiting nest sites in natural tree cavities or provided nest boxes. This difference in climate adaptation, in turn, matches a number of differences in life‐history traits. Collared flycatchers (i.e. the more ‘aggressive’ species) lay slightly smaller clutches of eggs, and their nestlings have higher begging rates (Qvarnström et al. 2007) and grow faster when conditions are favourable (Qvarnström et al. 2005, 2009), but experience higher mortality rates when food availability declines (Qvarnström et al. 2009; Veen et al. 2010). In most of these studies, we experimentally swapped nestlings between nests, that is we artificially created mixed species stepsibling broods, which allowed us to disentangle between differences in genetic or environmental effects. That pied flycatchers are less sensitive to food availability and that adult pied flycatchers provide a seasonally more stable nest environment for their offspring (Qvarnström et al. 2009) suggest that pied flycatchers can escape competition from collared flycatchers by breeding in less preferred habitat patches with a lower but broader peak of food availability. A recent comparison across several populations of pied flycatchers detected large geographical variation in the proportion of the most important food resource, caterpillar larvae, provided to nestlings (Burger et al. 2012). The broader overall habitat use of pied flycatchers (as a consequence of their broader breeding distribution) is hence associated with a pre‐existing flexibility in the use of food type that is yet another advantage when faced with competition against collared flycatchers.

Collared flycatchers started to colonize the Swedish island of Öland approximately 50 years ago, giving us a rare opportunity to study the ongoing processes of character displacement rather than inferring processes from patterns. Differences in climate adaptation between the two *Ficedula* species set the stage for the outcome of competitive interaction at secondary contact. Competitive exclusion from the preferred breeding habitats of pied flycatchers is mainly driven by the failure of 1‐year‐old pied flycatchers to establish breeding territories as the density of collared flycatchers increases (Vallin et al. 2012a). In the large Central European contact zone, collared flycatchers are found in greater numbers in warmer lowland areas, whereas pied flycatchers are more common in colder subalpine zones (Sætre et al. 1999a; Sætre et al. 1999b). Thus, the outcome of competitive interactions between the two species appears to follow a predictable pattern where some of the differences that have evolved in allopatry are strengthened in sympatry. The still ongoing ecological character displacements in the form of timing of breeding and habitat use (Vallin et al. 2012b) have direct effects on prezygotic reproductive isolation between the two species (Fig. 3, manuscript *in prep*). Moreover, hybrid nestlings appear to use an intermediate growth strategy and thereby have an environment‐dependent survival chance (Vallin et al. 2013), which has the potential to affect the likelihood of gene flow under different environmental conditions (Fig. 3). Thus, the flycatcher system demonstrates how allopatric divergence in climate adaptations can lead to asymmetry in competitive abilities and tolerance of harsh environmental conditions, which in turn lead to fast build‐up of spatial and temporal isolation at secondary contact. Young species that experience a similar climate at secondary contact may be selected to converge in habitat use and timing of breeding. Competition‐driven augmentation of differences that originate from adaptation to different climatic conditions experienced in geographically isolated populations of such young species, as shown in the flycatchers, may therefore, in general, play an important role to ensure the progression of speciation.

## Climate‐driven divergence in sexual display traits and assortative mating

### Climate and sexual selection

While there are several studies relating climate adaptation to prezygotic isolation mediated by spatial and temporal segregation, climate‐driven divergence in sexual display traits remains relatively unexplored. Sexual selection is generally acknowledged as an important evolutionary force driving population divergence (West‐Eberhard 1983; Price 1998; Panhuis et al. 2001; Edwards et al. 2005; Ritchie 2007; Kraaijeveld et al. 2011) but is often considered in isolation from natural selection in the context of speciation (reviewed by Maan and Seehausen 2011). However, theoretical models and empirical studies on sensory exploitation are an exception to this rule, where the central assumption is that the competing sex evolves display traits that exploit a pre‐existing bias in the sensory system of the choosy sex (reviewed by Boughman et al. 2002). These pre‐existing biases in the sensory system of the choosy sex evolve in response to patterns of natural selection, for example, associated with food types (Kolm et al. 2012). Adapting to different ecological environments may therefore lead to divergence in sensory systems and corresponding exploiting sexual display traits, which in turn leads to assortative mating.

The condition‐dependent nature of sexual display traits is rarely explored in theoretical models on speciation because divergence in mate preference is considered unlikely when females are selected to prefer males with the largest expression of the display trait (but see van Doorn et al. 2009). Temperature may have a strong impact on sexual selection as shown in superb fairy‐wrens (*Malurus cyaneus*; Cuckburn et al. 2008) and affect the intensity of display traits such as plumage in American redstarts (*Setophaga ruticilla*; Reudink et al. 2015) and calling rate in amphibians (Llusia et al. 2013). Male display traits could then often diverge in level of expression in geographical isolated populations, but it is unclear why corresponding preferences should diverge. Why would not females of both populations prefer males with the highest expression of the sexual signal at secondary contact? Different relationships between the expression of display traits and fitness depending on environmental conditions could lead to a corresponding divergent selection on mate preferences (Robinson et al. 2012). As climate adaptation is likely to have profound effect on the physiology of animals, the relationship between the expression of sexual traits and fitness may change depending on altered pleiotropic effects. Sexually selected signals based on variation in melanin‐based coloration often correlate with physiology (Roulin 2004), and these relationships can be environmentally dependent (Roulin et al. 2008) arising through pleiotropic effects of genes regulating both the synthesis of melanin coloration and other physiological traits (Ducrest et al. 2008; Roulin and Ducrest 2013). Melanin‐based sexual signals are therefore good candidates for mediating population assortative mating based on divergent climate adaptations. Climate‐induced changes in demography may affect the level of sexual selection in different populations (Kaneshiro 1989), which in turn can lead to evolutionary losses and gains of different sexual signals and thereby result in assortative mating. Furthermore, temperature can affect primary sexual characters and sperm competition as shown in insects (e.g. Berger et al. 2011; Gräzer 2013), which also has the potential to lead to reproductive isolation between populations adapting to different climates. There is generally limited empirical evidence for species assortative mating based on climate‐driven divergence in sexual display traits.

### Climate, sexual display traits and fitness in *Ficedula* flycatchers

The relationship between reproductive performance and sexual display in *Ficedula* flycatchers depends on both climatic conditions and interspecific interactions. Both species are sexually dimorphic in their plumage coloration during the breeding season. Females are dull grey‐brown and relatively alike, but males differ in several secondary sexual characteristics. Plumage trait variation is especially wide in pied flycatchers. The most conspicuous coloration trait in male pied flycatchers is the melanin‐based dorsal coloration that varies from completely brown to black (Drost 1936; Lundberg and Alatalo 1992), whereas collared flycatchers are always black on their dorsal side. Male collared flycatchers exhibit a distinctive white collar and conspicuous white ornamental patches on their forehead and wings (Cramp and Simmons 2006), while the size and shape of the forehead and wing patches in pied flycatchers vary more extensively (Laaksonen et al. 2015).

The highly variable dorsal coloration in pied flycatchers is subject to sexual selection such that black males have a mating advantage in allopatric populations (Lundberg and Alatalo 1992), while female pied flycatchers prefer brown males in the European hybrid zone (Sætre et al. 1997). Different eumelanin phenotypes in male pied flycatchers appear adapted to breed in different temperatures as judged from different reproductive outputs in different temperatures. The reproductive output of black males is the highest when it is cold during the egg‐laying phase but warm during the period when nestlings are fed (Sirkiä et al. 2010). Fluctuations in environmental conditions therefore maintain heritable within‐population phenotypic variation in plumage coloration where temperature‐dependent relative breeding success predicts the interannual change in the proportions of male colour phenotypes (Sirkiä et al. 2013). Black pied flycatcher males seem to be relatively more active when the spring is cold (Ilyina and Ivankina 2001), and swapping experiments reveal that it is the foster parent's melanin coloration that predicts reproductive success during the nestling phase. In low temperatures, foster offspring of black males were lighter than those raised by brown males, but the opposite was true when the temperature was relatively high during the nestling phase (Järvistö et al. 2015). The physiological and genomic basis for the link between variation in climate adaptation and melanin coloration in pied flycatchers remains unknown but is likely to be mediated by pleiotropic effects of genes regulating the synthesis of melanin coloration and other physiological traits as mentioned above.

The most extensively studied sexually selected plumage trait in collared flycatchers is the white forehead patch that males display in territorial disputes and when courting females. Males with relatively large forehead patches are dominant in territorial disputes (Pärt and Qvarnström 1997; Qvarnström 1997), are more likely to become polygynously mated (Gustafsson et al. 1995) and are less likely to lose paternity (Sheldon and Ellegren 1999). An experimental manipulation of the size of forehead patches showed that female preference for this trait varied seasonally according to predictable changes in the relative reproductive performance of large‐patched males (Qvarnström et al. 2000). A similar seasonal relationship between male forehead patch size and reproductive performance was found within the group of polygynously mated males (Qvarnström 2003). This predictable seasonal‐dependent relationship between forehead patch size and reproductive performance is likely driven by a difference in resource allocation between parental care and aggression (Qvarnström 1997, 1999). The relative reproductive performance of males with large forehead patches also fluctuates between years and can be predicted based on weather conditions. Small‐patched males perform better in years with high levels of rainfall (Robinson et al. 2012), which generally is associated with a high risk that offspring starve or freeze to death (e.g. Siikamäki 1996). Thus, males with relatively small forehead patches are subordinate but less sensitive to harsh conditions.

### Reproductive character displacement in *Ficedula* flycatchers

As in the case of ecological character displacement, the socially subordinate species responds stronger to sympatric conditions in terms of reproductive character displacement. In sympatry, male pied dorsal coloration becomes dull brown with less reflectance in UV, and the white patches on the forehead and wing become smaller, while the amount of white in outer retrixes becomes larger (Laaksonen et al. 2015; Sirkiä et al. 2015). The phenotypic differentiation between the populations (*P*_ST_) is higher than that in neutral genetic variation (*F*_ST_), indicating that pied flycatchers have diverged from collared flycatchers simultaneously in all these plumage traits in the area of secondary contact (Lehtonen et al. 2009) and that gene flow from the contact areas may affect coloration around the wide distribution of the species (Laaksonen et al. 2015). Dull coloration reduces interspecific male–male aggression (Sætre et al. 1993), and brown male pied flycatchers are allowed to settle closer to resident male collared flycatchers than black male pied flycatchers (Alatalo et al. 1994; Vallin et al. 2012b). Reduced aggression from collared flycatchers is also associated with relatively higher breeding success of brown than of black males in woodlots where collared flycatchers are present (Vallin et al. 2012b). Two not mutually exclusive hypotheses have been suggested to explain reduced aggression directed to relatively brown male pied flycatchers. First, dull coloration may mimic female coloration, thereby reducing male aggression (Sætre et al. 1993). The brown male phenotype of pied flycatchers has even been suggested to particularly mimic heterospecific females rather than intraspecific females (Calhim et al. 2014). Second, relatively brown males settle in comparatively low‐quality microhabitat also when breeding in areas without collared flycatchers, suggesting that a pre‐existing difference in microhabitat use reduces the intensity of competition with collared flycatchers (Vallin et al. 2012b). As a side effect of being more likely to breed in areas with many collared flycatchers, relatively brown males experience a higher risk of hybridization in the young Swedish hybrid zone (Vallin et al. 2012b). Competition between heterospecific males is hence the main driving force leading to fast reproductive character displacement (Vallin et al. 2012b). However, these findings do not rule out the possibility of reinforcement acting in parallel at a slower rate. In the old Central European hybrid zone, pied flycatcher females have indeed been found to prefer brown males over black ones (Sætre et al. 1997), which should reduce the risk of making mate choice errors.

Flycatchers provide an example of a study system where climatic adaptations and divergence by character displacement are closely linked. Brown male pied flycatchers are more sensitive to harsh environmental conditions, which is in line with the findings of most other studies on melanin coloration (e.g. Almasi et al. 2008; Ducrest et al. 2008; Roulin et al. 2008). The phenotype with lower degree of melanin coloration is best adapted to breed in an environment that is constantly warm during the breeding season, which matches the climatic requirements of the dominant collared flycatcher. We suggest that life‐history adaptations and sexually selected traits co‐evolve in the two flycatcher species and that these evolutionary processes have been affected by periods of repeated glaciations and interglacial intervals. Pleiotropic effects of genes regulating the synthesis of melanins may be one of the key links between climatic adaptations and divergence in eumelanin‐based plumage coloration, which is further suggested to be linked to other intercorrelated plumage characteristics (Sirkiä et al. 2015). Wiley et al. (2005) also raised the possibility of a link between variation in plumage traits and variation and developmental rate. This idea was based on two observations. First, 1‐year‐old male collared flycatchers display delayed plumage maturation with smaller forehead patch size, smaller wing patch size, less black dorsal coloration and more white on the outer tail feathers as compared to older male collared flycatchers. Second, the phenotype of collared flycatchers with such delayed plumage maturation is similar (and overlapping) to the plumage phenotype of pied flycatchers (that grow more slowly at the nestling stage).

The relationship between climate tolerance and aggressive behaviour/dominance signalling appears to differ in the two flycatcher species. This may, in turn, have important consequences for the feedback loops between evolution in allopatry and sympatry (Fig. 2). The most dominant, black pied flycatchers with relatively large white patches appear relatively better adapted to northern climate than brown males (Sirkiä et al. 2010, 2013), while the most dominant collared flycatchers, that is with large forehead patch sizes, instead are not as well adapted to northern climate conditions than males with small forehead patches (Robinson et al. 2012). Divergence in dominance signalling is therefore associated with convergence in climate requirements (i.e. in areas where the two species co‐occur under similar climatic conditions). Gene flow from sympatric areas has been suggested to affect the proportion of brown males throughout the breeding distribution of pied flycatchers (Laaksonen et al. 2015), hence possibly leading to convergence in climate requirements of the two species on a larger geographical scale (Fig. 2). Species divergence in plumage is directly linked with prezygotic reproductive isolation in the form of species assortative mating (e.g. Sætre et al. 1997; Wiley et al. 2005; Fig. 3) and with postzygotic reproductive isolation by reducing the sexual attractiveness of hybrid males (Svedin et al. 2008; Fig. 3). In birds, females constitute the heterogametic sex (ZW), while males are homogametic (ZZ). Female heterogamy is assumed to favour the build‐up of linkage disequilibrium between male display traits and female preferences facilitating maintenance of prezygotic isolation under gene flow (Reeve and Pfennig 2003; Kirkpatrick and Hall 2004). Sex chromosomes are furthermore well‐known hot spots for harbouring genetic incompatibilities (Qvarnström and Bailey 2009). Both plumage traits (Sætre et al. 2003) and species recognition (Sæther et al. 2007) are linked to the Z chromosome in flycatchers. A detailed study on the genomic landscape of species divergence between these two moreover revealed a much higher level of species divergence of the Z chromosome as compared to the autosomes (Ellegren et al. 2012). The role of divergence in sexually selected display traits in the speciation process has hence been extensively studied in *Ficedula* flycatchers. Plumage coloration (i.e. degree of blackness and size of the white patches) is subject to a whole suite of selection pressures including sexual selection operating within each species, effects of climate adaptation within each species, competition between the two species and reinforcement resulting from selection against hybridization.

Processes of sexual selection where females prefer males displaying superior condition and thereby ability to provide material or genetic benefits (Andersson 1994) are, as mentioned above, rarely assumed to lead to the disruptive selection regime needed for evolution of assortative mating. In other words, females may be faced with a trade‐off between species and mate‐quality recognition (Pfennig 1999), where sexual selection operating within each species actually counteracts the evolution of species assortative mating. Studies on *Ficedula* flycatchers illustrate that this ‘problem’ can be solved when the relationship between male display traits and fitness (and hence male ability to provide material and/or genetic benefits) varies in relation to external conditions. Relatively black male pied flycatchers are better adapted to the harsh climate experienced in populations that are geographically isolated from collared flycatchers. By contrast, brown male pied flycatchers are better adapted to competition with male collared flycatchers and experience relatively higher fitness in regions of secondary contact with collared flycatchers. Therefore, female pied flycatchers do not experience a trade‐off between species and mate‐quality recognition and should evolve a preference for the brown morphs in secondary contact zones. Whether this situation is exclusive for flycatchers or generally occurring remains an open question.

## Climate adaptation and the build‐up of genetic incompatibilities

### Links between ecology and build‐up of genetic incompatibilities remain largely unknown

Populations that evolve in allopatry for a substantial amount of time are likely to accumulate different mutations that will result in genetic incompatibilities at secondary contact. Genetic incompatibilities often result in loss of hybrid fitness, hybrid sterility or even hybrid inviability (Coyne and Orr 2004). According to the Dobzhansky–Muller model, incompatibilities arise because certain new alleles brought together in one genome through hybridization have never been previously ‘tested’ together by selection and may simply not work together or be incompatible with the other species' genomic background, as those genes have not co‐evolved (Dobzhansky 1936; Müller 1940). Incompatibilities are thought to accumulate following a ‘snowball effect’, where genetic incompatibilities increase exponentially over time, as the number of possible incompatible combinations increases dramatically with each new mutation fixed in each of the isolated populations (Turelli et al. 2001). Genetic differences can accumulate by drift, divergent natural and sexual selection, as a result of sexual conflict (Arnqvist and Rowe 2005), or because of meiotic drives and the evolution of corresponding suppressors (Frank 1991). According to the mutation‐order hypothesis, similar environmental conditions can result in fixation of different mutations, because different genetic pathways can lead to the same adaptation (Schluter and Conte 2009). Selection for divergence as a direct consequence of adaptation to different conditions such as climate should result in faster evolution of postzygotic isolation, resulting more often in populations that already have reduced (intrinsic) hybrid fitness at secondary contact, which in turn would favour the evolution of enhanced prezygotic isolation through reinforcement processes (Howard 1993).

Studies directly linking ecological pressures to the evolution of genetic incompatibilities are scarce, and this is also true in relation to climate or thermal adaptation (Keller and Seehausen 2012). This is because the role of ecology is often studied very early in the speciation continuum, between divergent populations or incipient species (in allopatry or sympatry) that are still perfectly interfertile (Schluter and Conte 2009). Intrinsic postzygotic isolation, on the other hand, is most obvious (and mainly studied) between perfectly distinct species that often diverged long ago and accumulated many barriers, thus making it difficult to infer the order in which each of the barriers appeared, and particularly what caused the apparition of genetic incompatibilities (Orr 1995; Turelli et al. 2001; Rice et al. 2011). Intrinsic postzygotic isolation has been reported between species adapted to different environments (e.g. MacNair and Christie 1983; Kruuk et al. 1999; Rieseberg et al. 1999; Rogers and Bernatchez 2006; Fuller 2008; Lowry et al. 2008). The genes or genomic regions involved in intrinsic incompatibilities have been identified in several of those systems, but whether the same genetic regions underlay adaptations is in most cases unknown. Only one study directly showed that the same genes (or closely linked genes) that underlay the ecological adaptation also cause incompatibilities in hybrids, that is a gene causing copper resistance (or closely linked genes) in *Mimulus guttatus* (MacNair and Christie 1983). In an experimental study, several strains of yeasts were exposed to selection in different environments (two suboptimal environments in terms of high salinity and low glucose) over 500 generations (Dettman et al. 2007). This allowed a careful study of the direct causes of adaptation to harsh environments at the genetic level. Stronger divergent selection resulted in higher levels of dysfunction in hybrid growth rate and meiosis (Dettman et al. 2007). Thus, apart from a few examples, knowledge about the role of ecological adaptation in the build‐up of genetic incompatibilities is generally lacking.

We are not aware of any study showing that genes underlying divergent climate adaptation cause genetic incompatibility between species. However, spermatogenesis is particularly sensitive to perturbation in gene expression (Sun et al. 2004) and external conditions (Wu and Davis 1993). Temperature is likely to influence the genes involved in spermatogenesis, and divergent climates may lead to different rates of evolution of genes important for regulatory functions. A mismatch between mitochondrial and nuclear genomes, a potentially common form of hybrid incompatibility, might regularly trigger the origin of hybrid sterility (Burton et al. 2006). Numerous mitochondrial genes depend on nuclear gene products to perform essential cell replication and maintenance functions (Burton and Barreto 2012), and there is thus strong selection for co‐evolution between nuclear and mitochondrial genes, which might be decoupled in hybrid individuals. Several processes involving mitochondrial genes are temperature‐sensitive and might evolve differently in different climates (Dowling et al. 2008). As sperm are ‘propelled’ by the energy produced by their mitochondria (Froman and Feltmann 1998), a mismatch between mitochondria and nuclear genomes is particularly likely to affect hybrid sperm performance.

### Genetic incompatibilities in birds and *Ficedula* flycatchers

In birds, genetic incompatibilities generally evolve more slowly than in other taxa (Price and Bouvier 2002; Price 2007). Many species are formed through premating barriers long before the build‐up of genetic incompatibilities. In the classical textbook example of the Darwin finches, several species seem to be perfectly compatible when brought together on the same island by rare climatic events (Grant et al. 1996). Furthermore, the recently sequenced genomes of all 15 species of the Darwin finches show extensive past and present introgressive hybridization (Lamichhaney et al. 2015). In a survey on the evolution of postzygotic incompatibilities in birds, Price and Bouvier (2002) showed that 92% of crosses between species of different genera result in some loss of hybrid fitness, while this happens in only 38‐50% of the crosses between species of the same genus. Following Haldane's rule (Glossary; see Box 2), the first sex to be affected is the female (the heterogametic sex in birds), and sterility of both sexes occurs before females lose viability. A loss of fertility appears between species showing on average 14% of sequence divergence (in mitochondria), which corresponds to 7 million years, while inviability evolves after 25% or 12.5 million years of divergence. Note that most of those crosses were executed in captivity, and even though most avian species are thought to have evolved in allopatry (Price 2007), very little is known about genetic incompatibilities between naturally hybridizing birds, and even less about the factors leading to those incompatibilities.

The *Ficedula* flycatchers show a high level of postzygotic isolation for being such ‘young’ avian species. Indeed, all female hybrids are sterile (Svedin et al. 2008) and hybrid males experience strongly reduced fertility, caused by impaired spermatogenesis and resulting in extreme rates of hatching failures in their nests and high infidelity by their females (Ålund et al. 2013). As the two species diverged less than one million years ago (Nadachowska‐Brzyska et al. 2013), and their genomes are very similar (Ellegren et al. 2012), this level of intrinsic postzygotic isolation is high. It is thus rather unlikely that drift alone leads to this fast rate of accumulation of incompatibilities, and we suggest that adaptation to different climates in allopatry could have caused important genetic changes affecting hybrid fertility.

We are planning to explore the physiological and genomic background to the observed trade‐off between competitive ability (i.e. signalled by plumage traits) and tolerance to harsh environment in *Ficedula* flycatchers by, for example, measuring metabolic rate and performing genomewide association mapping. These are necessary steps to explore whether divergence in climate adaptation relates to the build‐up of genetic incompatibilities. To test this, we would first need to establish the genomic basis of key phenotypic traits associated with differences in climate adaptation and then test for possible pleiotropic effects of the detected genes (or of genes in close linkage) on hybrid dysfunction. Using genomewide association methods based on a custom‐made 50 k SNP array, we recently identified three markers of moderate effect for clutch size in collared flycatchers (Husby et al. 2015), that is one of the life‐history traits that differ between the two species. As this method depends on the presence of within‐species variation, it will not capture possible fixed genetic differences between the two species (which would require admixture mapping and gene expression studies). However, all genes belonging to networks of genes involved in incompatibilities need not be fixed between species but could instead be maintained variable within each species (or one of them) through mutation selection balance. It is premature to conclude any possible role for climate adaptation in the build‐up of genetic incompatibilities between the two *Ficedula* flycatchers.

The role of climate adaptation in the build‐up of genetic incompatibilities between species remains, in general, largely unexplored as relevant technology has only recently become available for application to natural systems. We may, however, speculate that divergent climate adaptation should be particularly likely to cause genetic incompatibilities between species. The main reason for this prediction is that temperature is known to have such a large effect on the biochemistry of living organisms (e.g. Kingsolver 2009) and thereby central life‐history traits. Disturbed epistatic interactions (i.e. due to lack of a co‐evolutionary history) between genes underlying divergent climate adaptation may therefore have fundamental effects on hybrid fertility and viability.

## Conclusions and future directions

We reviewed the evidence of climate adaption in the build‐up of reproductive isolation between populations with a particular emphasis on studies using collared and pied flycatchers as a model system for speciation research. There are several solid examples of differences in climate adaptations causing prezygotic isolation in the scientific literature. These studies typically show that divergent climate adaptation reduces the likelihood that individuals from different species mate as they are separated in space or timing of breeding (e.g. Martin 2001; Lowry and Willis 2010; Bertolo et al. 2011; Kahilainen et al. 2014). Our studies on the flycatcher system are different in the aspect that we investigate links between adaptations gained in allopatry and competitive interactions at secondary contact. There are a number of differences in life‐history traits and in sexual display traits between the two *Ficedula* flycatchers that affect the relative performance of these two species under different climatic and social conditions. These differences likely reflect an evolutionary history of cycles with adaption to different climates under allopatric conditions (i.e. pied flycatchers have a more northern breeding distribution) and periods of competition and hybridization at secondary contact augmenting some of the pre‐existing differences (Fig. 2) but also leading to convergence in other traits.

In short, pied flycatchers are less dominant and therefore experience a disadvantage in competition over nest sites at secondary contact, but collared flycatchers are more sensitive to harsh environmental conditions (food shortage and rainfall) reflecting a more narrow niche use (e.g. Qvarnström et al. 2005, 2009; Veen et al. 2010). At the within‐species level, robustness to variation in climate (i.e. temperature and rainfall) can be predicted based on male plumage display traits in terms of the level of melanin (Sirkiä et al. 2010, 2013) and size of white patches (Robinson et al. 2012). The asymmetry in competitive ability between the two flycatcher species (i.e. partly caused by adaptation to different climates) leads to ecological and reproductive character displacements in pied flycatchers. In the young Swedish hybrid zone, pied flycatchers are progressively displaced into poorer habitat and later onset of breeding (Vallin et al. 2012b). In addition, brown plumage coloration in male pied flycatchers is favoured by selection in the presence of collared flycatchers (Vallin et al. 2012b). Hence, competition and selection against hybridization at secondary contact augment some of the central pre‐existing differences between the two species. However, other traits converge at secondary contact, including climate adaptation. Thus, divergent climate adaptation appears to have set the stage for divergence in traits that promote reproductive isolation between the two *Ficedula* species. Once these traits have diverged (e.g. habitat use and plumage coloration), divergent climate adaptation *per se* likely becomes redundant for keeping the two species segregated. How ecological forces act on population pairs that undergo divergence under both allopatric and sympatric conditions during their speciation process in general needs more scientific attention and could illuminate the long‐term role of climate in speciation processes.

For studies of speciation, hybrid zones offer excellent possibilities to pinpoint the traits that influence assortative mating, hybrid fitness and interspecific gene flow (e.g. Barton and Hewitt 1985; Harrison and Rand 1989; Arnold 1997). One particularly interesting aspect of hybrid zone dynamics is how different sources of reproductive isolation interact. We have studied many different classes of reproductive barriers in the flycatchers (Fig. 3), which open up several interesting pathways for future research. For example, we can investigate how the evolutionary processes leading to different sources of reproductive isolation interact, for example, through competitive interaction and through reinforcement (Howard 1993) or reinforcement‐like processes (Lorch and Servedio 2007). Another major aim is to investigate the underlying genetic constitution of species‐specific adaptations that influence reproductive isolation (i.e. related to ecological performance and mating patterns) and ultimately test whether there are direct links to the build‐up of genetic incompatibility. Answering these kinds of questions would bring us closer to understanding interactions between different mechanisms driving speciation.

Studying processes of adaptation, which are morphologically cryptic and possible links to the build‐up of typically slowly arising reproductive barrier (e.g. genetic incompatibilities), has become more feasible than ever before. We see the presentation of the genomic divergence landscape of *Ficedula* flycatchers (Ellegren et al. 2012) as an existing starting point rather than an endpoint for research aiming to understand the flycatcher speciation process. In general, the possibility to study patterns of genomewide divergence opens up fantastic new pathways for research within the field of speciation. It is now possible to study how mechanisms of divergence such as genetic drift, natural and sexual selection/sexual conflict, and meiotic drive interact with properties of the genome itself such as genomewide variation in recombination rates. On the one hand, low levels of recombination shelter co‐adapted gene complexes from being dissembled, which should be particularly important for speciation under gene flow. On the other hand, genomic regions with low recombination rates are expected to be at a higher risk of accumulating deleterious mutations due to Muller's ratchet and hitchhiking with beneficial mutations (e.g. Charlesworth et al. 1987; Rice 1987). Butlin (2005) therefore suggested that genomic regions reducing recombination between diverging populations without having similar effects within populations (e.g. chromosomal inversions) should be particularly likely to be involved in speciation. However, the influence of recombination rates on the speciation process remains largely unexplored. We suggest that one interesting new research question would be to investigate whether selection acting directly on the rate of recombination could vary across geographical contexts of the speciation process. Periods of primary or secondary contact should favour evolution of restricted recombination rates as competition over resources and mates should favour specialization into different strategies and hence avoidance of homogenizing effects of gene flow on co‐evolved gene complexes. Allopatric conditions should instead favour increased rates of recombination through reduced rate of accumulation of deleterious mutations and increased rate of adaptive adjustments to harsh and fluctuating environmental conditions at the edge of species breeding ranges.
